# Supplementary material for: Predicting visual function by interpreting a neuronal wiring diagram
Source: Nature. 2024 Oct 2;634(8032):113–23. doi: 10.1038/s41586-024-07953-5 (PMC11446822; doi:10.1038/s41586-024-07953-5)
Supplement: Supplementary file 6 — CRF and ERF predictions for individual TmY4 and TmY9 cells. Analogous to Supplementary Data 3, but for TmY target types. Shown are the top four monosynaptic pathways, the strongest pathway passing through each of the top ten intermediary types (ranking from Extended Data Fig. 7), and the trisynaptic pathway Tm1–TmY–Dm3–TmY (see the section entitled Prediction of spatial normalization). [file 41586_2024_7953_MOESM6_ESM.zip › DataS4/TmY9qΓèÑ/720575940616321465.pdf]

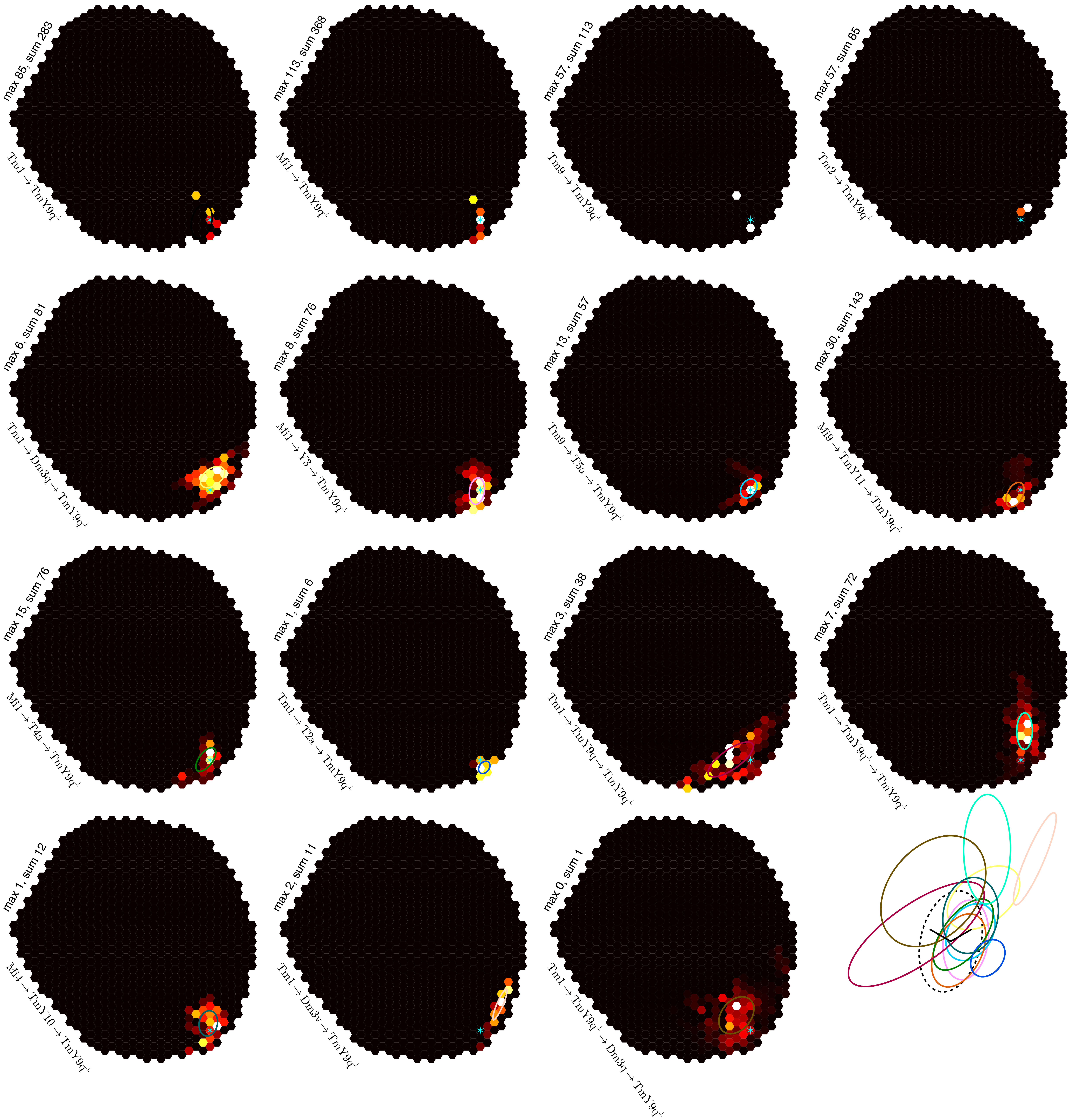
max 85, sum 283
Tm1 → TmY9q⊥
max 113, sum 368
Mi1 → TmY9q⊥
max 57, sum 113
Tm9 → TmY9q⊥
max 57, sum 85
Tm2 → TmY9q⊥
max 6, sum 81
Tm1 → Dm3q → TmY9q⊥
max 8, sum 76
Mi1 → Y3 → TmY9q⊥
max 13, sum 57
Tm9 → T5a → TmY9q⊥
max 30, sum 143
Mi9 → TmY11 → TmY9q⊥
max 15, sum 76
Mi1 → T4a → TmY9q⊥
max 1, sum 6
Tm1 → T2a → TmY9q⊥
max 3, sum 38
Tm1 → TmY9q⊥ → TmY9q⊥
max 7, sum 72
Tm1 → TmY9q⊥ → TmY9q⊥
max 1, sum 12
Mi4 → TmY10 → TmY9q⊥
max 2, sum 11
Tm1 → Dm3v → TmY9q⊥
max 0, sum 1
Tm1 → TmY9q⊥ → Dm3q → TmY9q⊥
